# In silico vaccine design and epitope mapping of New Delhi metallo-beta-lactamase (NDM): an immunoinformatics approach

**DOI:** 10.1186/s12859-021-04378-z

**Published:** 2021-09-25

**Authors:** Matin Fathollahi, Anwar Fathollahi, Hamid Motamedi, Jale Moradi, Amirhooshang Alvandi, Ramin Abiri

**Affiliations:** 1 Department of Microbiology, School of Medicine, Kermanshah University of Medical Sciences, Kermanshah, Iran; 2 Department of Immunology, School of Medicine, Shahid Beheshti University of Medical Sciences, Tehran, Iran; 3 Medical Technology Research Center, Health Technology Institute, Kermanshah University of Medical Sciences, Kermanshah, Iran; 4 Fertility and Infertility Research Center, Health Technology Institute, Kermanshah University of Medical Sciences, Kermanshah, Iran

**Keywords:** New Delhi metallo-beta-lactamase, In silico, Vaccine design, Immunoinformatics, Multi-epitope based vaccine

## Abstract

**Background:**

Antibiotic resistance is a global health crisis. The adage that “prevention is better than cure” is especially true regarding antibiotic resistance because the resistance appears and spreads much faster than the production of new antibiotics. Vaccination is an important strategy to fight infectious agents; however, this strategy has not attracted sufficient attention in antibiotic resistance prevention. New Delhi metallo-beta-lactamase (NDM) confers resistance to many beta-lactamases, including important carbapenems like imipenem. Our goal in this study is to use an immunoinformatics approach to develop a vaccine that can elicit strong and specific immune responses against NDMs that prevent the development of antibiotic-resistant bacteria.

**Results:**

In this study, 2194 NDM sequences were aligned to obtain a conserved sequence. One continuous B cell epitope and three T cell CD4^+^ epitopes were selected from NDMs conserved sequence. Epitope conservancy for B cell and HLA-DR, HLA-DQ, and HLA-DP epitopes was 100.00%, 99.82%, 99.41%, and 99.86%, respectively, and population coverage of MHC II epitopes for the world was 99.91%. Permutation of the four epitope fragments resulted in 24 different peptides, of which 6 peptides were selected after toxicity, allergenicity, and antigenicity assessment. After primary vaccine design, only one vaccine sequence with the highest similarity with discontinuous B cell epitope in NDMs was selected. The final vaccine can bind to various Toll-like receptors (TLRs). The prediction implied that the vaccine would be stable with a good half-life. An immune simulation performed by the C-IMMSIM server predicted that two doses of vaccine injection can induce a strong immune response to NDMs. Finally, the GC-Content of the vaccine was designed very similar to *E. coli* K12.

**Conclusions:**

In this study, immunoinformatics strategies were used to design a vaccine against different NDM variants that could produce an effective immune response against this antibiotic-resistant factor.

**Supplementary Information:**

The online version contains supplementary material available at 10.1186/s12859-021-04378-z.

## Background

Antibiotic resistance that is a direct consequence of the overwhelming use of antibiotics is one of the biggest issues that threaten global health. The World Health Organization (WHO), the Food and Agriculture Organization (FAO), and the World Organization for Animal Health (OIE) have considered antibiotic resistance as one of the three declared priority issues for joint action [1]. The United Nations (UN) also reported that the death toll due to antibiotic resistance is at least 700,000 deaths a year in 2019, which will be raised to 10 million deaths by 2050. Furthermore, economic damage due to uncontrolled antimicrobial resistance may lead to an economic shock similar to the 2008–2009 global financial crisis, which can result in more poverty and inequality [2].

The currently available strategies to avoid or prevent antibiotics resistance suggested by the Centers for Disease Control and Prevention (CDC) as the “Four Core Actions to Prevent Antibiotic Resistance” includes preventing infections and preventing the spread of resistance, tracking antibiotic-resistant infections, avoiding unnecessary uses of antibiotics in humans and animals and developing new drugs and diagnostic tests [3]. Designing a vaccine to prevent antibiotic resistance falls into the first class of strategies suggested by the CDC.

As β-lactamases are very important in conferring resistance to many essential antibiotics, there are numerous published papers about their prevalence, single nucleotide polymorphisms, and their inhibitors. These enzymes led to antibiotic resistance in a wide range of human pathogens through the inactivation of β-lactam antibiotics which posed serious challenges for the treatment of patients. The mechanism of action of beta-lactamase is through hydrolysis of the β-lactam ring in the structure of β-lactam antibiotics. The β-lactam ring is the core structure of β-lactam antibiotics, therefore its hydrolysis leads to the inactivation of these antibiotics [4, 5]. Normally, β-lactamases are divided into two groups according to the characteristics of the active site. In the first group, the acyl-enzyme contains serine in the active site (classes A, C, and D) while in the second group the hydrolytic reaction is facilitated by one or two zinc ions (class B). The second group is called metallo-β-lactamases (MBLs) [6, 7], and New Delhi metallo-beta-lactamase (NDM) is categorized in this class. The first member of NDMs (NDM-1) was reported in 2009 from a Swedish patient traveling to New Delhi, India with the bacterium *Klebsiella pneumoniae*. It hydrolyzes a wide range of β-lactam antibiotics including penicillins, late-generation cephalosporins, and carbapenems, except monobactams [8]. These enzymes are found in a variety of bacterial pathogens including *K. pneumoniae, Escherichia coli*, *Acinetobacter baumannii*, *Enterobacter cloacae*, *Providencia rettgeri*, and *Morganella morganii*. As different plasmids carrying the *bla*NDM gene, these enzymes can be transmitted between microorganisms in different ways including inter-strain, inter-species, and inter-genus [9].

Immunoinformatics approaches have revolutionized vaccine design and development by increasing the quality of empirical studies. In-silico methods help to focus exclusively on well-analyzed and rational vaccine candidates. For example, Immunoinformatics approaches can help to determine conserved sequences. Conserved sequences are sequences that are similar or identical between different isoforms of a protein or nucleic acid sequences from various sources. The conserved sequences are important targets for vaccine design as they allow production vaccines effective against different isoforms of the target protein to cover a large range of potential targets and reduce the probability of resistance occurrence [10].

Furthermore, another important consideration during the design of a vaccine is the determination of antigenicity, immunogenicity, and allergenicity of the candidate epitopes. An Antigen is a molecular structure that can bind specifically to antibodies, B cell receptors, or T cell receptors; while immunogens are antigens that can induce either or both humoral or cell-mediated immune responses. Some antigens cannot induce immune responses at the first encounter, therefore they are not immunogenic. Allergens are immunogens or antigens that induce a pathogenic immune response due to over-activation of the immune system [11, 12]. Immunoinformatics approaches can predict the most available and immunogenic epitopes that have the minimum possibility of toxicity, allergenicity, and cross-reactivity to host antigens [13].

Another advantage of bioinformatics approaches is the possibility of “codon optimization” Amino acids are commonly expressed by synonymous codons. In various organisms, these synonymous codons are used with unequal frequency, which is called codon bias (CB). Codon optimization results in similar GC content. Each organism has its specific codons usage and therefore specific GC content; therefore, to have a maximum expression of a cloned protein sequence in an organism, the sequence must have synonymous codons with the host [9, 10]. We used E. coli K12 as the host, so all the codons must be optimized according to the E. coli K12 genome.

**Fig. 1:**
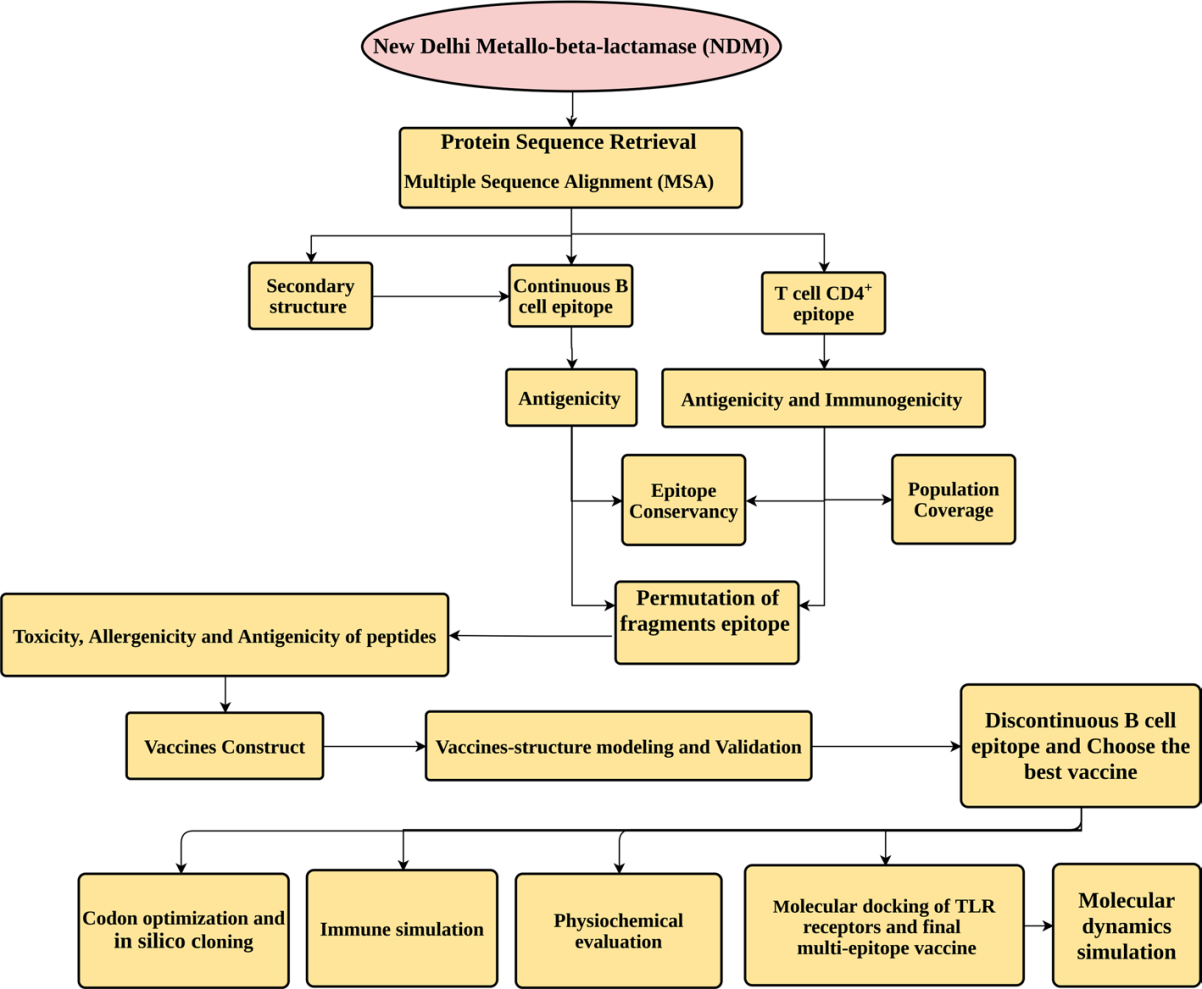
Schematic workflow of in silico design of New Delhi metallo-beta-lactamase (NDM) vaccine

The adage that “prevention is better than cure” is especially true regarding antibiotic resistance because the resistance is introduced and spread much faster than the production of new antibiotics [14, 15]. Vaccination is an important strategy to fight infectious agents; however, this strategy has not attracted sufficient attention in antibiotics resistance prevention. Our goal in this study is to use an immunoinformatics approach (Fig. 1) to develop a vaccine that can elicit strong and specific immune responses against NDMs that prevent the development of antibiotic-resistant bacteria.

## Results

### Conserved protein sequence

The final conserved sequence contains 229 amino acids. The conserved sequence obtained from the multiple sequence alignment (MSA) is presented below (for more details regarding MSA methodology refer to the “1-Protein Sequence Retrieval” in the methods section).

GDQRFGDLVFRQLAPNVWQHTSYLDMPGFGAVASNGLIVRDGGRVLVVDTAWTDDQTAQILNWIKQEINLPVALAVVTHAHQDKMGGMDALHAAGIATYANALSNQLAPQEGMVAAQHSLTFAANGWVEPATAPNFGPLKVFYPGPGHTSDNITVGIDGTDIAFGGCLIKDSKAKSLGNLGDADTEHYAASARAFGAAFPKASMIVMSHSAPDSRAAITHTARMADKLR.

### Secondary structure

**Fig. 2:**
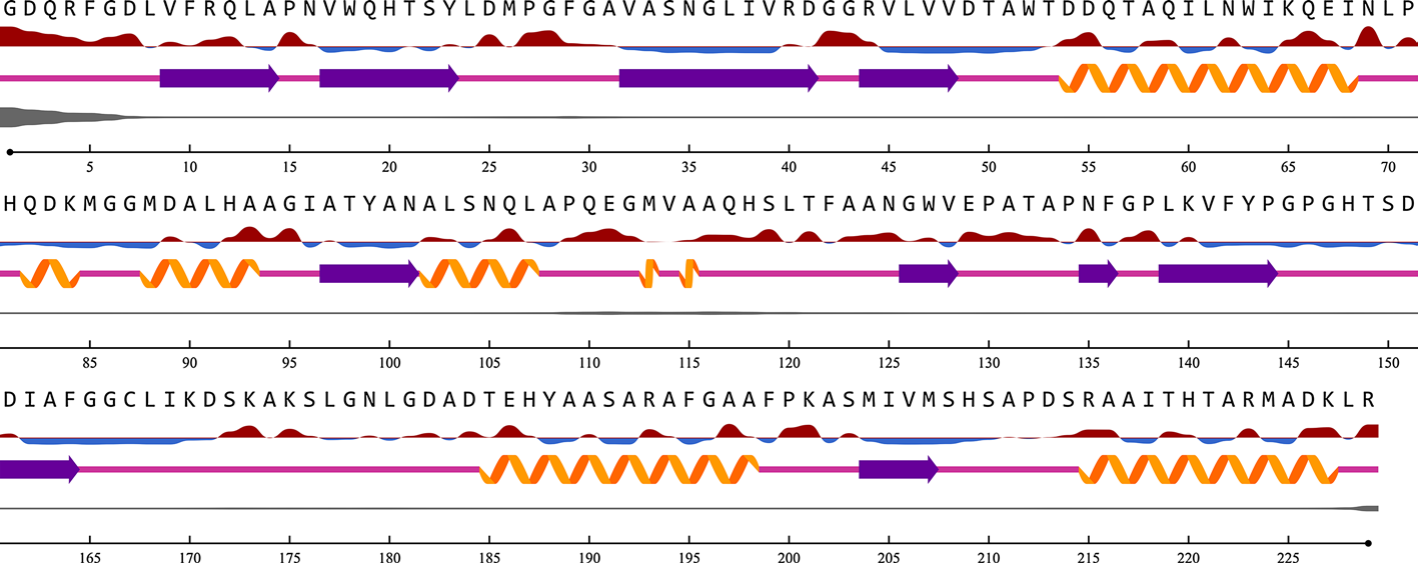
The first line represents the amino acids constituting the conserved sequence. The second line represents the relative surface accessibility (RSA), in which a red curve at the top of the line denotes the residue is exposed and the blue at the bottom of the line indicates buried residues. The third line represents the Secondary Structure, including the strand (purple thick arrow), the coil (pink straight lines), and the helix (orange spiral shapes). The fourth line represents disordered residues in the structure; the thickened regions indicate the presence of disordered residues. The fifth line shows the sequence number of residues

The secondary structure of the conserved sequence obtained from the NetSurfP-2.0 Server has approximately 74.23% coil, 27.51% strand, and 25.76% helix. About the first six amino acids in the conserved sequence were disordered residues. Considering the threshold at 25% for Relative Solvent Accessibility (RSA), approximately 45.41% of amino acids contain RSA (Fig. 2).

### Continuous B cell epitope

Continuous B cell epitopes were detected from all three servers LBtope, ABCpred, and SVMtrip, and the top five epitopes are listed in Table 1. The antigenicity of these epitopes was also assessed by the VaxiJen v3.0 server. In the top five epitopes of the LBtope server, the peptide GGCLIKDSKAKSLGN (165–179) with a score of 0.95000785 is in the first rank and has an antigenicity with a probability of 66%. Although this peptide is not among the 5 top epitopes ranked by ABCpred, however, it has an acceptable score to be considered as a candidate epitope. Indeed, the calculated score for this epitope is 0.73, which is much higher than the defined threshold for this server that is 0.51.

**Table 1:** Continuous B cell epitope obtained from LBtope, SVMtrip and ABCpred servers

	Rank	Location	Epitope	Score	Antigenicity (probability)
LBtope	1	165–179	GGCLIKDSKAKSLGN	0.95000785	Antigen (66%)
	2	164–178	FGGCLIKDSKAKSLG	0.94638595	Antigen (66%)
	3	168–182	LIKDSKAKSLGNLGD	0.76900064	Non-antigen (66%)
	4	65–79	KQEINLPVALAVVTH	0.76639088	Non-antigen (66%)
	5	166–180	GCLIKDSKAKSLGNL	0.74369164	Non-antigen (100%)
SVMTriP	1	174–189	AKSLGNLGDADTEHYA	1	Antigen (66%)
	2	156–171	GIDGTDIAFGGCLIKD	0.949	Antigen (100%)
	3	37–52	LIVRDGGRVLVVDTAW	0.763	Non-antigen (66%)
	4	201–216	KASMIVMSHSAPDSRA	0.74	Non-antigen (100%)
	5	62–77	NWIKQEINLPVALAVV	0.733	Non-antigen (66%)
ABCpred	1	20–35	HTSYLDMPGFGAVASN	0.91	Antigen (100%)
	2	211–226	APDSRAAITHTARMAD	0.89	Non-antigen (66%)
	3	203–218	SMIVMSHSAPDSRAAI	0.88	Antigen (66%)
	4	79–94	HAHQDKMGGMDALHAA	0.85	Non-antigen (66%)
	5	35–50	NGLIVRDGGRVLVVDT	0.84	Non-antigen (66%)

### T cell CD4^+^ epitope

The MHC-II alleles that are abundant in at least one European country were selected. Regarding this including criteria, twelve HLA-DR alleles, five HLA-DQ haplotypes, and two HLA-DP haplotypes were chosen (Table 2).

**Table 2:** Haplotypes and alleles that were abundant in one or more European countries

HLA-DR	HLA-DQ	HLA-DP
DRB1*01:01	DQA1*01:01/DQB1*05:01	DPA1*01/DPB1*04:01
DRB1*03:01	DQA1*01:02/DQB1*06:02	DPA1*01:03/DPB1*02:01
DRB1*04:01	DQA1*03:01/DQB1*03:02	
DRB1*04:04	DQA1*05:01/DQB1*02:01	
DRB1*04:05	DQA1*05:01/DQB1*03:01	
DRB1*07:01		
DRB1*08:01		
DRB1*09:01		
DRB1*11:01		
DRB1*11:04		
DRB1*13:01		
DRB1*15:01		

**Fig. 3:**
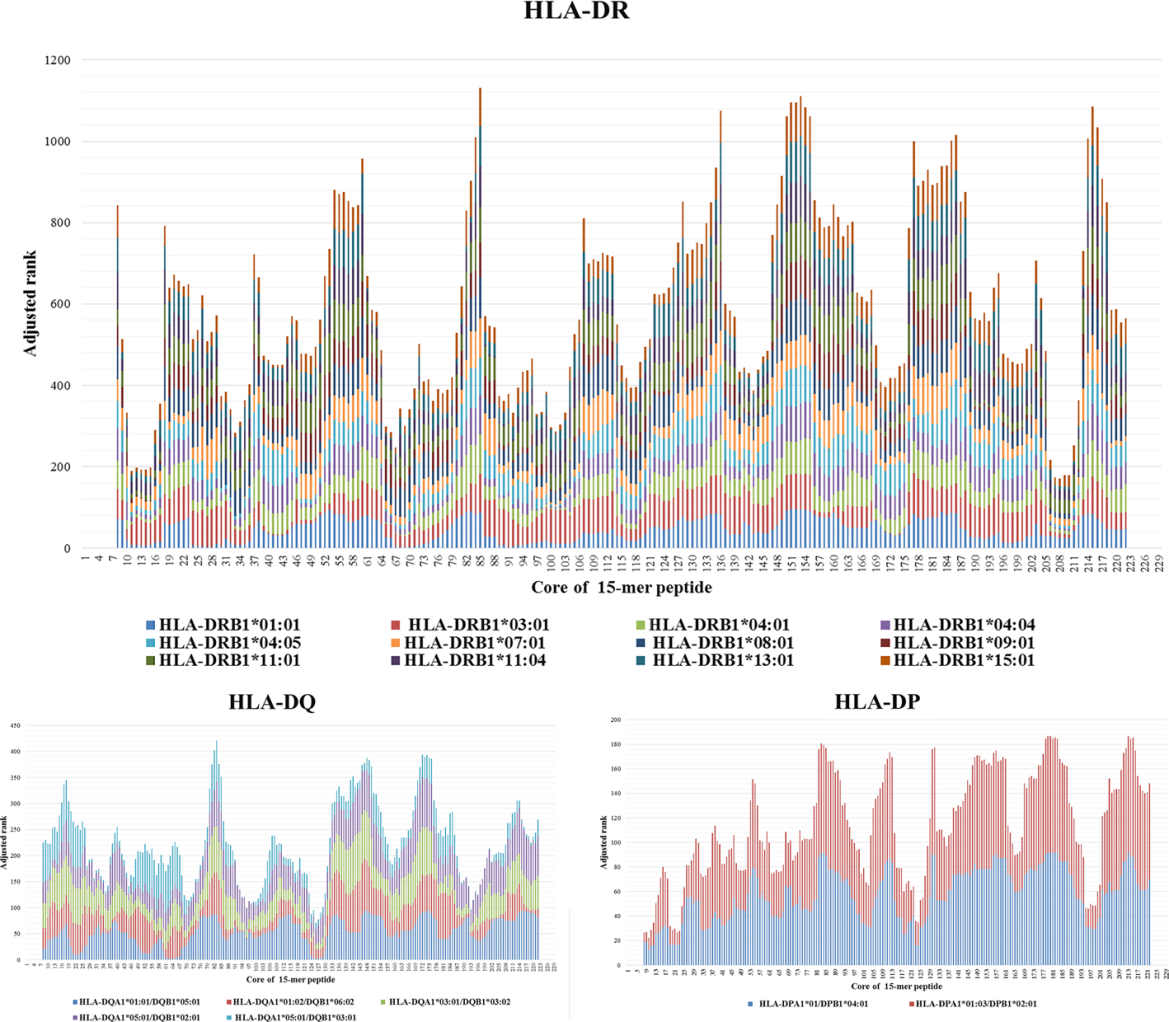
The results of prediction of HLA-DR, HLA-DQ, and HLA-DP epitopes by the IEDB Consensus method; the lower adjusted rank indicates good binder epitopes. The epitope length was defined as 15 residues (15 mer) and each peptide is named by a number equals to the median values of the respective peptide sequence number; e.g. the 1–15 peptide is named core of peptide 8. In HLA-DR epitopes, peptide cores 208, 207, 210, 209, and 11, in HLA-DQ epitopes peptide cores 193, 98, 96, 192, and 97 and in HLA-DP epitopes 10, 22, 8, 9, and 23 demonstrated the lowest adjusted rank which implies that they are top good binder epitopes

In the Consensus method (for more details regarding the IEDB Consensus method refer to the “4-T cell CD4 + epitope” in the methods section), the lower the adjusted rank epitope means the better binding of the epitopes to MHC alleles. The results of the Consensus method for HLA-DQ, and HLA-DP haplotypes, and HLA-DR alleles are depicted in Fig. 3, and the top five of each chart are summarized in Table 3. In addition, the antigenicity of each of the top five peptides was calculated by the VaxiJen v3.0 server and is depicted in the last column of Table 3. The epitopes that were identified by the VaxiJen v3.0 server as non-antigen was not selected as the final vaccine epitopes even if they are among the five top IEDB Consensus rank. Finally, PKASMIVMSHSAPDS (200–214) epitope for HLA-DR alleles, EHYAASARAFGAAFP (186–200) epitope for HLA-DQ haplotypes, and DQRFGDLVFRQLAPN (2–16) epitope for HLA-DP haplotypes were selected. The top five immunogenic epitopes predicted by the IEDB are listed in Table 4. The GDLVFRQLAPNVWQH epitope (6–20) with first rank immunogenicity with a score of 49.57 overlaps with the selective epitope HLA-DQP in 11 amino acids and the KASMIVMSHSAPDSR epitope (201–215) that sat at the fourth rank of immunogenicity with a score of 42.21 in IEDB, overlaps with HLA-DR epitope in 14 amino acids.

**Table 3:** Top five HLA-DR, HLA-DQ, and HLA-DP epitopes

	Rank	Location	Epitope	Antigenicity (probability)
HLA-DR	1	201–2015	KASMIVMSHSAPDSR	Non-antigen (66%)
	2	200–214	PKASMIVMSHSAPDS	Antigen (66%)
	3	203–217	SMIVMSHSAPDSRAA	Non-antigen (66%)
	4	202–216	ASMIVMSHSAPDSRA	Non-antigen (66%)
	5	4–18	RFGDLVFRQLAPNVW	Non-antigen (66%)
HLA-DQ	1	186–200	EHYAASARAFGAAFP	Antigen (100%)
	2	91–105	LHAAGIATYANALSN	Antigen (66%)
	3	89–103	DALHAAGIATYANAL	Antigen (66%)
	4	185–199	TEHYAASARAFGAAF	Antigen (100%)
	5	90–104	ALHAAGIATYANALS	Antigen (100%)
HLA-DP	1	3–17	QRFGDLVFRQLAPNV	Non-antigen (66%)
	2	15–29	PNVWQHTSYLDMPGF	Non-antigen (66%)
	3	1–15	GDQRFGDLVFRQLAP	Non-antigen (66%)
	4	2–16	DQRFGDLVFRQLAPN	Antigen (66%)
	5	16–30	NVWQHTSYLDMPGFG	Antigen (66%)

**Table 4:** Top five peptide with immunogenicity for CD4^+^ T cells

Rank	Peptide	Location	Combined score*
1	GDLVFRQLAPNVWQH	6–20	49.57224
2	LNWIKQEINLPVALA	61–75	46.54788
3	MPGFGAVASNGLIVR	26–40	45.15712
4	KASMIVMSHSAPDSR	201–215	42.21464
5	IATYANALSNQLAPQ	96–110	41.36456

*For more details regarding combined score refer to the “5-Prediction of antigenicity and immunogenicity of peptide fragments” in the methods section

### Epitope conservancy

The selected B cell recognizing epitope, GGCLIKDSKAKSLGN (165–179) was conserved in all the 2194 retrieved initial sequences. The selected HLA-DR binding epitope, PKASMIVMSHSAPDS (200–214), was 100% identical in 2190 initial sequences, and only four sequences had identities less than 100%. These four sequences are WP_109791213.1 with a 93.33% identity, OES48450.1, BBE58699.1 and APY22234.1 with 80% identity. The selected HLA-DQ binding epitope, EHYAASARAFGAAFP (186–200), was 100% identical with 2181 initial sequences and 93.33 identity with thirteen other sequences, including SYX53208.1, OES48450.1, AGS56768.1, QCQ28762.1, QCQ28543.1, WP_032495384.1, AWL48936.1, ASB81781.1, WP_063860858.1, AWM64865.1, AMO51458.1, WP_094009810.1 and WP_123002102.1. Selected HLA-DP recognizing epitope is DQRFGDLVFRQLAPN (2–16) with 100% identity with 2191 initial sequences and less identity for three other sequences including 93.33% identity with WP_152315467.1, and 26.67% identity with QID22101.1 and AQT38377.1 (Table 5).

**Table 5:** Epitope conservancy of B cell, HLA-DR, HLA-DQ, and HLA-DP epitopes

Epitope name	Epitope sequence	Epitope length	Percent of protein sequence matches at identity < = 100%	Minimum identity (%)	Maximum identity (%)
B cell	GGCLIKDSKAKSLGN	15	100.00% (2194/2194)	100.00	100.00
DR	PKASMIVMSHSAPDS	15	99.82% (2190/2194)	80.00	100.00
DQ	EHYAASARAFGAAFP	15	99.41% (2181/2194)	93.33	100.00
DP	DQRFGDLVFRQLAPN	15	99.86% (2191/2194)	26.67	100.00

### Population coverage

World population coverage (for more details regarding Population coverage refer to the “7-Population coverage” in the methods section) was 99.91% for all selected alleles of HLA-DR, HLA-DQ, and HLA-DP, while the coverage rate was 100% for Europe and North America. Other regions (except South Africa) had more than 98.5% population coverage and only South Africa had population coverage of less than 50% (Table 6).

**Table 6:** Predicting population coverage of MHC II epitopes

Population/area	Coverage (%)
Central Africa	99.86
Central America	99.90
East Africa	99.96
East Asia	98.84
Europe	100.00
North Africa	99.79
North America	100.00
Northeast Asia	99.78
Oceania	99.92
South Africa	47.29
South America	99.91
South Asia	99.94
Southeast Asia	98.68
Southwest Asia	99.55
West Africa	99.88
West Indies	98.68
World	99.91

### Toxicity, allergenicity and antigenicity of the peptides

Toxicity, allergenicity, and antigenicity of these 24 peptides were evaluated as it is required for any potential therapeutic protein.

Nine out of the initial 24 peptides were found to be non-toxic and 13 out of the 24 peptides were identified with a 100% antigen probability. Eleven peptides were ignored due to inefficient antigenicity, of which 10 peptides were estimated with only a 66% antigen probability, and one was estimated non-antigenic with a 66% probability (Table 7). Finally, even though AllergenFP detected none of the peptides as an allergen, 10 out of the 24 peptides were identified as an allergen by AllerTOP v.2.0 server.

**Table 7:** Evaluation of toxicity, allergenicity and antigenicity 24 permutation-derived peptides

Rank	Peptide names	Combination of epitopes	Toxicity	Antigenicity (%)	Allergenicity (AllerTOP v. 2.0)	Allergenicity (AllergenFP v.1.0)
1	Pep1	B-DQ-DP-DR	0.014561407	Antigen (100%)	Non-allergen	Non-allergen
2	Pep2	B-DP-DQ-DR	0.020762188	Antigen (100%)	Non-allergen	Non-allergen
3	Pep3	B-DQ-DR-DP	0.026229585	Antigen (100%)	Non-allergen	Non-allergen
4	Pep4	B-DR-DP-DQ	0.035163384	Antigen (66%)	Non-allergen	Non-allergen
5	Pep5	B-DR-DQ-DP	0.035222273	Antigen (66%)	Non-allergen	Non-allergen
6	Pep6	B-DP-DR-DQ	0.04321611	Antigen (100%)	Non-allergen	Non-allergen
7	Pep7	DQ-B-DP-DR	0.043704998	Antigen (66%)	Allergen	Non-Allergen
8	Pep8	DP-DQ-B-DR	0.044408895	Antigen (100%)	Non-allergen	Non-allergen
9	Pep9	DP-B-DQ-DR	0.04492618	Antigen (100%)	Non-allergen	Non-allergen
10	Pep10	DQ-B-DR-DP	0.07196537	Non-antigen (66%)	Allergen	Non-allergen
11	Pep11	DQ-DP-B-DR	0.07311195	Antigen (66%)	Allergen	Non-allergen
12	Pep12	DR-B-DQ-DP	0.07872102	Antigen (100%)	Non-allergen	Non-allergen
13	Pep13	DP-B-DR-DQ	0.089660764	Antigen (66%)	Allergen	Non-allergen
14	Pep14	DR-B-DP-DQ	0.0922846	Antigen (100%)	Non-allergen	Non-allergen
15	Pep15	DP-DR-B-DQ	0.09429422	Antigen (66%)	Allergen	Non-allergen
16	Pep16	DQ-DR-B-DP	0.096130505	Antigen (66%)	Allergen	Non-allergen
17	Pep17	DR-DQ-B-DP	0.09999384	Antigen (100%)	Non-allergen	Non-allergen
18	Pep18	DR-DP-B-DQ	0.1686433	Antigen (66%)	Non-allergen	Non-allergen
19	Pep19	DP-DR-DQ-B	0.33977464	Antigen (100%)	Allergen	Non-allergen
20	Pep20	DP-DQ-DR-B	0.35059118	Antigen (100%)	Allergen	Non-allergen
21	Pep21	DQ-DR-DP-B	0.39058733	Antigen (66%)	Allergen	Non-allergen
22	Pep22	DR-DQ-DP-B	0.40825927	Antigen (100%)	Non-allergen	Non-allergen
23	Pep23	DQ-DP-DR-B	0.45443133	Antigen (66%)	Allergen	Non-allergen
24	Pep24	DR-DP-DQ-B	0.5206262	Antigen (100%)	Non-allergen	Non-allergen

Collectively, 6 peptides out of the 24 primary peptides were identified to have 100% antigenicity without toxicity and allergenicity. These peptides were selected and named Pep1, Pep2, Pep3, Pep6, Pep8, and Pep9.

### Vaccine-structure modeling and validation

The Pep1, Pep2, Pep3, Pep6, Pep8, and Pep9 peptides were linked by EAAAK linker to Cholera toxin subunit B (CTB) adjuvant at their N-terminal to create vaccines named 1, 2, 3, 4, 5 and, 6, respectively.

The RaptorX server predicts 5 structures with the lowest root-mean-square deviation (RMSD) score for each peptide sequence. To further determine the best of those 5 structures, the MolProbity server was used. MolProbity server utilizes 3 criteria to compare the structures including favored rotamers, Ramachandran favored, and Rama-Z score. These 3 criteria were used to determine the best possible structure for the downstream steps in the study. In other words, for each one of the 6 candidate vaccines, one of the 5 structures was chosen as the optimum structure (Table 8). Favored rotamers and Ramachandran favored values are given as a percentage. The best structures were given the highest percentages and the ideal value is usually higher than 98%. The Rama-Z score value should be ideally between − 2 and + 2, i.e. abs (Z score) < 2. Finally, from the vaccine models listed in Table 8, vaccine 1 structure 2, vaccine 2 structure 3, vaccine 3 structure 2, vaccine 4 structure 5, vaccine 5 structure 3, and vaccine 6 structure 3, were selected based on optimum scores in the following four values: RMSD, Favored rotamers, Ramachandran favored and Rama-Z score.

**Table 8:** Evaluation of RMSD, favored rotamers, Ramachandran favored, and Z-score of six vaccine constructs

	Rank	RMSD (Å)	Favored rotamers (%)	Ramachandran favored (%)	Z-score
Vaccine 1	1	7.5107	96.23	93.56	− 2.32 ± 0.49
	2	6.9878	98.11	93.07	− 1.63 ± 0.53
	3	7.2056	99.37	92.08	0.80 ± 0.58
	4	8.2021	99.37	88.12	− 1.24 ± 0.57
	5	10.122	95.60	92.57	− 1.72 ± 0.52
Vaccine 2	1	7.3	98.74	94.06	0.73 ± 0.61
	2	8.1636	98.11	93.56	− 1.57 ± 0.54
	3	7.9784	100.00	97.03	0.68 ± 0.58
	4	8.018	97.48	94.55	0.05 ± 0.56
	5	8.2166	98.74	94.55	− 0.86 ± 0.55
Vaccine 3	1	6.4034	100.00	90.59	− 1.99 ± 0.52
	2	7.1167	100.00	94.55	0.49 ± 0.59
	3	7.7406	98.11	93.56	− 1.90 ± 0.54
	4	8.2312	98.74	95.05	0.22 ± 0.59
	5	9.4755	97.48	94.55	− 1.14 ± 0.55
Vaccine 4	1	8.0477	94.34	90.10	− 1.95 ± 0.54
	2	8.0874	98.74	94.55	− 2.82 ± 0.52
	3	9.6278	96.86	95.05	− 1.12 ± 0.55
	4	8.2284	96.86	95.05	0.71 ± 0.59
	5	10.131	98.74	95.54	− 0.72 ± 0.56
Vaccine 5	1	6.8903	98.11	92.57	0.28 ± 0.59
	2	7.2672	96.86	93.07	0.49 ± 0.60
	3	7.6564	98.74	96.53	− 1.14 ± 0.54
	4	8.7086	98.11	95.54	− 1.76 ± 0.55
	5	7.7784	97.48	95.05	0.56 ± 0.59
Vaccine 6	1	6.5636	96.23	92.08	− 0.32 ± 0.60
	2	7.2168	99.37	93.07	− 0.87 ± 0.55
	3	7.3298	99.37	96.04	0.18 ± 0.61
	4	7.4151	96.23	87.13	− 2.71 ± 0.52
	5	8.1497	96.86	92.08	− 1.39 ± 0.50

### Discontinuous B cell epitope

**Fig. 4:**
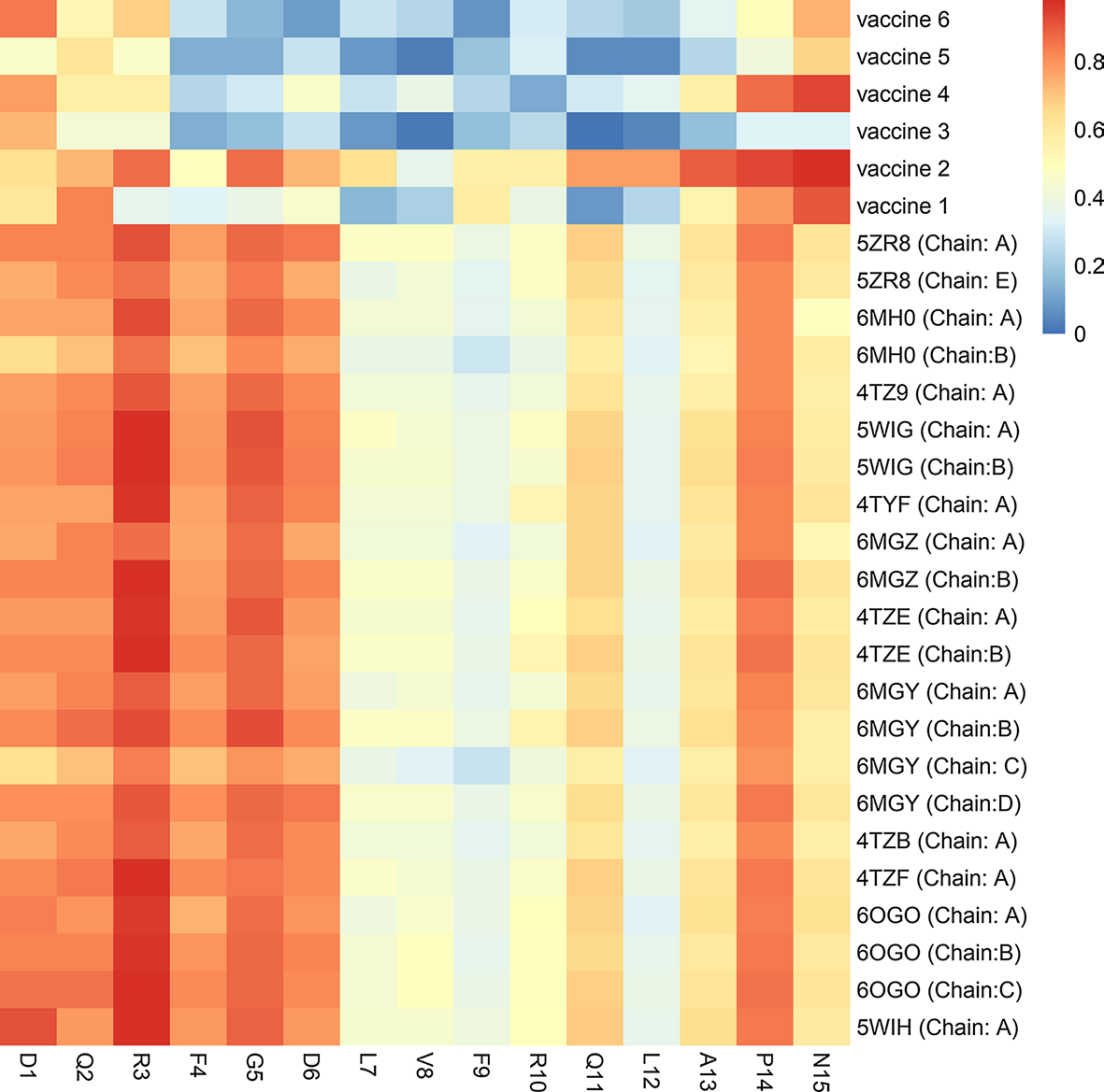
Heatmap visualization allows the comparison of the predicted discontinuous epitope in six candidate vaccine constructs with twelve selected NDM proteins. According to the heatmap gradient of colors scale, the vaccine 2 has the most similarity with the NDM proteins (Visualization was performed by R software, available from: https://www.R-project.org)

The discontinuous B cell epitopes of the six vaccine constructs and all of the existed chains of twelve NDM proteins retrieved from PDB were predicted by the ElliPro server (Table 9). Consequently, the discontinuous B cell epitopes from the vaccine constructs were compared with the twelve NDM proteins to identify discontinuous epitopes in the vaccine constructs that are identical or similar to the discontinuous epitopes in the NDM proteins. The analysis detected one sequence that was present only in vaccine construct 2 with high similarity to the DQRFGDLVFRQLAPN sequence in positions 42 to 57 of NDMs proteins. This common sequence had high-scoring amino acids for discontinuous B cell epitopes (Fig. 4).

**Table 9:** PDB ID of twelve NDM proteins used in discontinuous epitope prediction

PDB ID	Chains	Sequence length	Organism	Protein
5ZR8	A, E	246	*Escherichia coli*	NDM-1
6MH0	A, B	243	*Escherichia coli*	NDM-3
4TZ9	A	231	*Escherichia coli*	NDM-3
5WIG	A, B	230	*Klebsiella pneumoniae*	NDM-4
4TYF	A	231	*Escherichia coli*	NDM-4
6MGZ	A, B	243	*Klebsiella pneumoniae*	NDM-4
4TZE	A, B	231	*Escherichia coli*	NDM-5
6MGY	A, B, C, D	243	*Klebsiella pneumoniae*	NDM-5
4TZB	A	231	*Escherichia coli*	NDM-6
4TZF	A	231	*Escherichia coli*	NDM-8
6OGO	A, B, C	243	*Escherichia coli*	NDM-9
5WIH	A	230	*Escherichia coli*	NDM-12

### Molecular docking of multi-epitope vaccine with TLR receptors

**Fig. 5:**
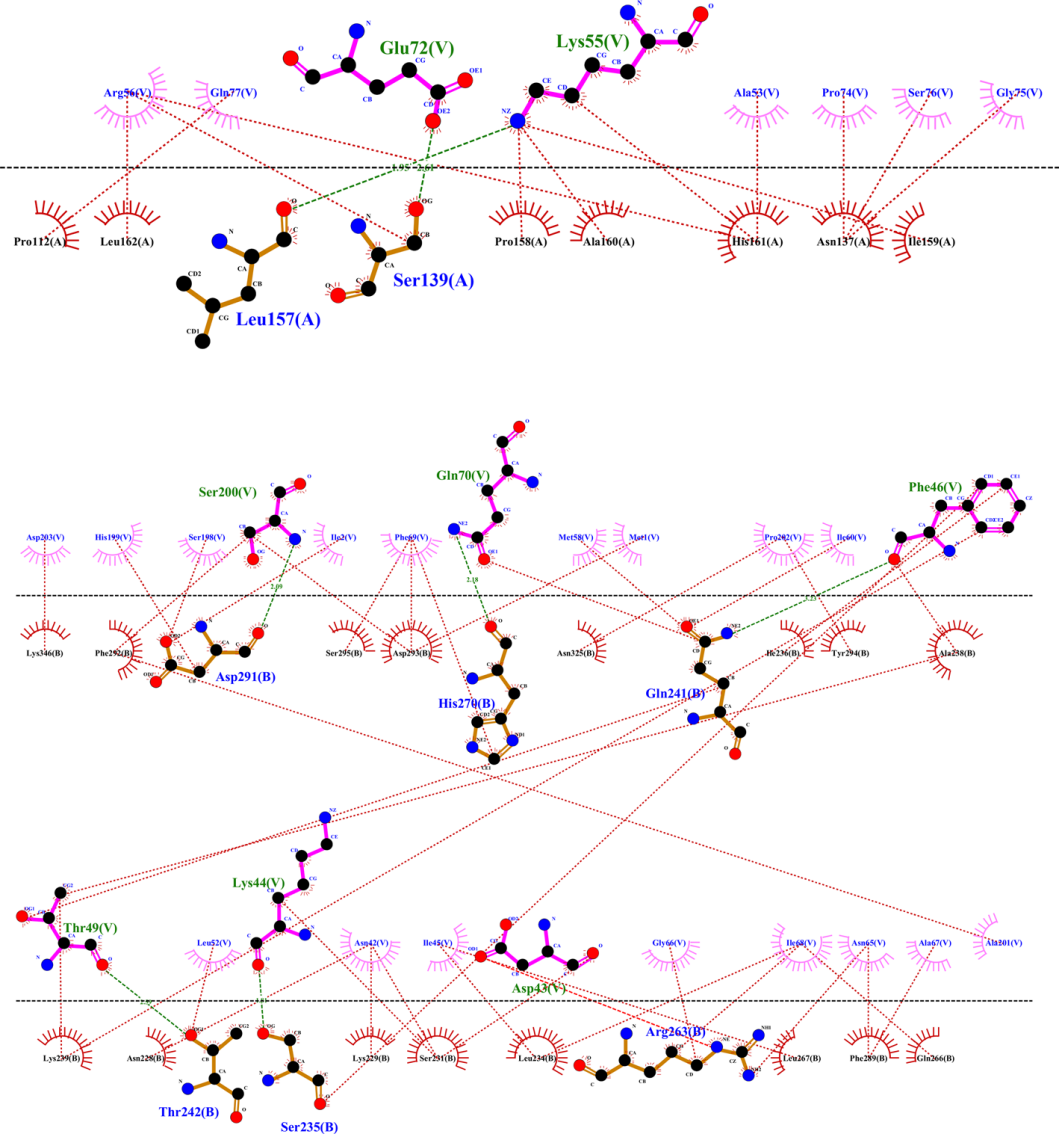
Interacting residues between docked vaccine (Chain V) with TLR1 (Chain A and Chain B). The green, red, and brick red dashed lines represent hydrogen bonds, salt bridges, and hydrophobic interactions, respectively. The hydrogen bonds and salt bridges are more important interactions in the complex. The Leu157 and Ser139 residues from chain A of TLR1 were bound to Lys55 and Glu72 residues of the vaccine by hydrogen bonds with bond lengths of 1.95 angstroms (Ǻ) and 2.61 Ǻ, respectively. The Asp291, His270, Gln241, Thr242 and Ser235 residues from the chain B of TLR1 are bound to the Ser200, Gln70, Phe46, Thr49 and Lys44 residues of the vaccine with hydrogen bonds with bond lengths of 2.09 Ǻ, 2.18 Ǻ, 3.23 Ǻ, 2.59 Ǻ and 1.81 Ǻ, respectively. In addition, the Arg263 residue from the chain B of TLR1 is bound to the Asp43 residue of the vaccine with the salt bridges interaction vaccine

Molecular docking of vaccine 2 construct with Toll-like receptors (TLRs) was performed by using the PatchDock server. The top ten vaccine 2 construct-TLRs docking results by this server was obtained. The Global Energy of the above-mentioned ten results was calculated by FireDock (Additional file 1: Table 1). Vaccine 2 construct interacted with TLR1 (PDB ID: 6nih) with a score of 15,210 and Global Energy − 34.76 kilocalories per mole (kcal/mol), with TLR1-TLR2 (PDB ID: 2z80) with a score of 14,090 and Global Energy − 39.00 kcal/mol and with two different states from TLR4 (PDB ID: 2Z62) with 14,908 and 13,056 scores and Global Energies − 35.78 kcal/mol and − 45.30 kcal/mol, respectively. In addition, binding free energy of the complexes calculated by MM/GBSA, and resulted in − 70.89, − 45.08, − 47.7, and − 31.6 kcal/mol, respectively. Hydrogen bonds, salt bridges, and hydrophobic interactions were obtained by the DIMPLOT program. The Vaccine complex with TLR1 (PDB ID: 6nih) is shown in Fig. 5 and the other complexes listed in Additional file 2: Fig. 1.

### Normal mode analysis (NMA)

**Fig. 6:**
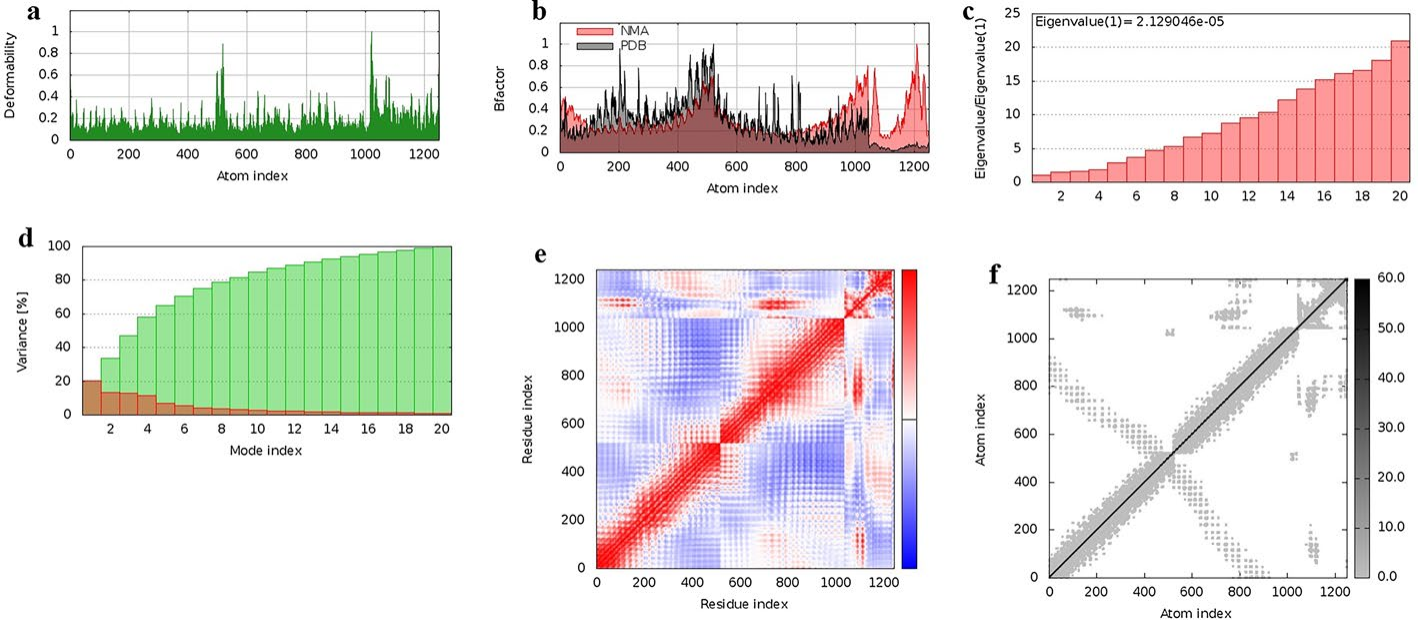
Normal mode analysis of the vaccine-TLR1 complex. The graphs represent **a** Deformability, **b** B-factor, **c** Eigenvalues, **d** Variance, **e** Covariance map **f** Elastic network (for more details regarding Normal mode analysis refer to the “13-Normal mode analysis” in the methods section)

Normal mode analysis (for more details regarding Normal mode analysis refer to the “13-Normal mode analysis” in the methods section) of the TLR1 vaccine complex (PDB ID: 6nih) is shown in Fig. 6, and the vaccine complex with TLR1-TLR2 (PDB ID: 2z80) and TLR4 (PDB ID: 2Z62) are also shown in the Additional file 3: Fig. 2. Areas of protein that have deformability are peaked in Fig. 6a. The B-factor graph shows the divergence of the complex in the PDB file with NMA that is depicted in Fig. 6b. The eigenvalue graph is also shown in Fig. 6c and its numerical value is equal to 2.129046e-05. Furthermore, the variance graph corresponding to the normal mode is shown in Fig. 6d. The covariance map in Fig. 6e shows pairs of residues with correlated motions in red, uncorrelated motions in white and anti-correlated motions in blue. Finally, the darker grays in Fig. 6f show rigid regions (stiffer springs) in the elastic network.

### Physiochemical evaluation of vaccine

Physicochemical properties obtained by the ProtParam tool. The simulation of the half-life in mammalian reticulocytes as in vitro environment was estimated to be 30 h, and in yeast and Escherichia coli, as in vivo environment, was estimated to be more than 20 and 10 h, respectively. Other calculated physicochemical properties included 21,842.04 Daltons for molecular weight, 9.08 for theoretical isoelectric point (pI), 75.74 for aliphatic index, − 0.195 for a grand average of hydropathicity (GRAVY), 32.32 for instability index. Furthermore, the number of residues with a negative charge (Asp + Glu) and positive charge (Arg + Lys) were determined 17 and 22, respectively. Finally, C975H1531N267O285S9 was obtained as the vaccine formula.

### Immune simulation

**Fig. 7:**
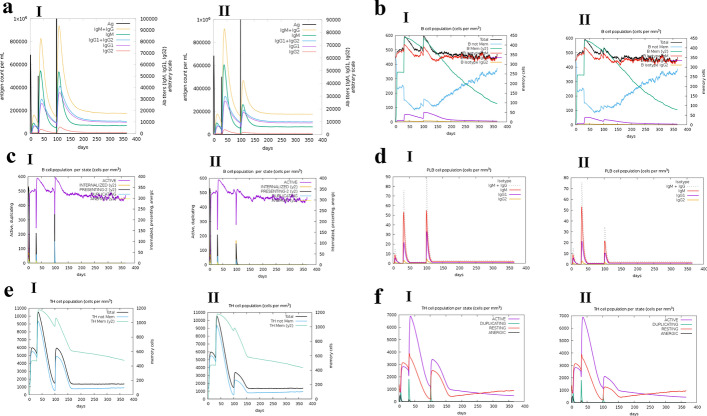
Immune simulation after two doses of vaccine introduction at first and 30th days and exposure to specific (ai-fi) or nonspecific (aii-fii) antigen on the 100th day. **a** antibody titer **b** B lymphocytes and sub-populations **c** B lymphocytes per state **d** Plasma B lymphocytes **e** T-helper lymphocytes **f** T-helper lymphocytes per state (for more details regarding Immune simulation refer to the “15-Immune simulation” in the methods section)

The immune simulation was performed by the C-ImmSim server which predicted that two doses of vaccine injection can induce a strong immune response to NDMs. Based on the evaluation of the immune response by the C-ImmSim server; a specific response regarding antibody titer after exposure to the specific antigen was much higher than the non-specific antigen (Fig. 7a). B-cell subpopulations including B-cell memory and Plasma B lymphocytes (PLB cells) also had a higher peak on the 100th day (Fig. 7b–d). In addition, the T helper cell population demonstrated a higher peak in the specific antigen group compared to the nonspecific antigen on the 100th day after injection of specific antigen (Fig. 7e–f).

### Codon-optimization and cloning

**Fig. 8:**
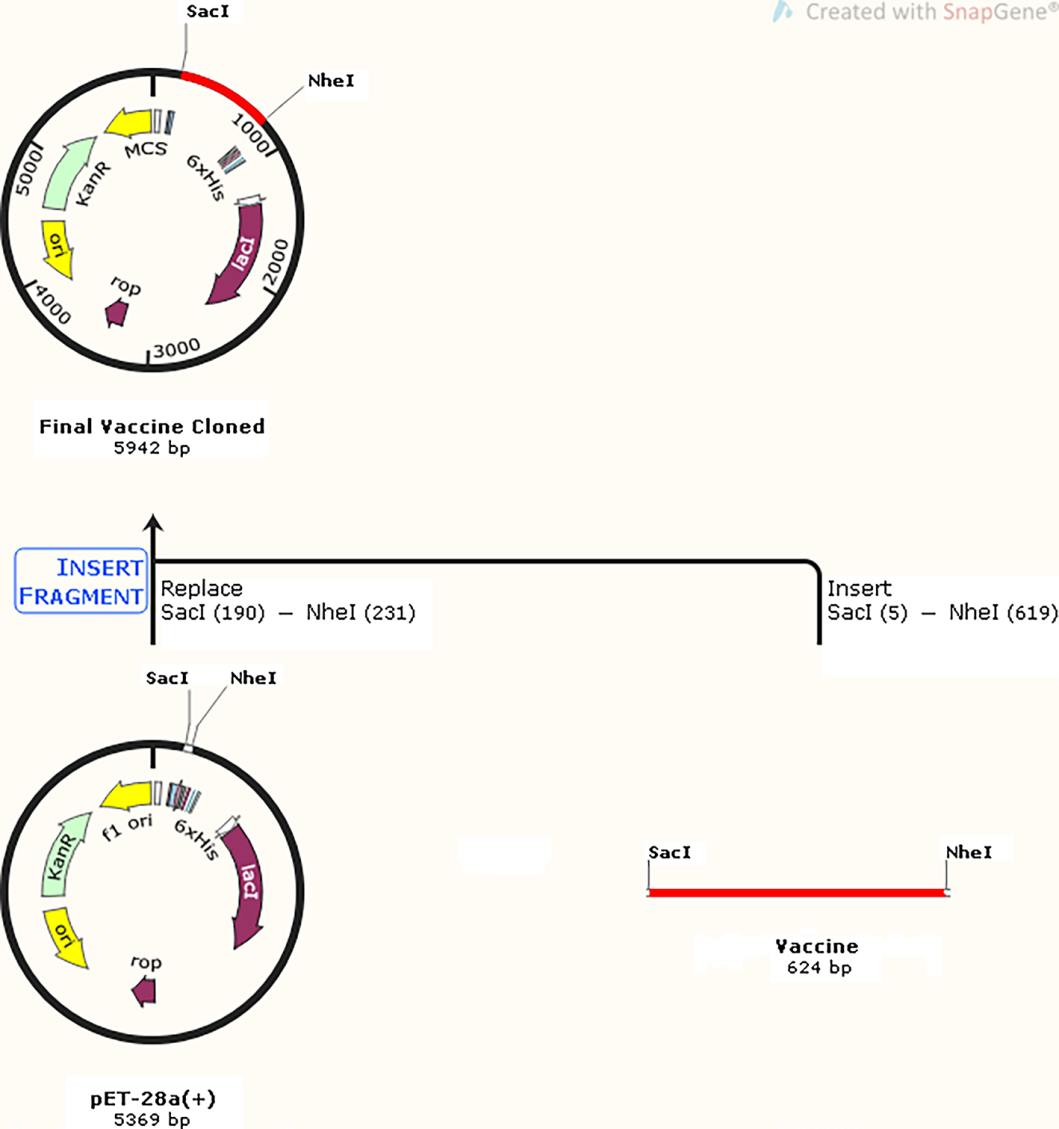
In silico cloning simulation. Schematic representation of the codon-optimized multi-epitope vaccine sequence (Red) insertion into pET-28a (+) expression vector (black) between Restriction sites SacI and NheI

The final vaccine was codon-optimized for *E. coli* strain K12 using the JCAT server. Finally, a DNA sequence of 612 nucleotides with CAI-Value of 1.0 and GC-Content of 51.14 was obtained, which is very close to the GC-Content of *E. coli* strain K12 with 50.73. In addition, the Restriction sites SacI (GAGCTC) and NheI (GCTAGC) were added to the N and C terminals of the final vaccine codon sequence, then this sequence was added into the pET-28a (+) vector by the SnapGene tool (Fig. 8).

## Discussion

Due to the widespread use of antibiotics, antibiotic-resistant bacteria are on the rise, and many of previously used antibiotics are ineffective now. The discovery of new antibiotics has failed to keep pace with the emergence of antibiotic-resistant pathogens. We are moving forward to a stage where we are no longer able to cure a large number of bacterial infections. NDMs are one of the most important antibiotic resistance-conferring enzymes that present in a wide range of human pathogens and their prevalence is increasing worldwide [16, 17]. Although vaccination is one of the best preventive approaches to fight infectious agents, no vaccine has yet been developed to prevent antibiotics resistance. We believe that producing a vaccine against bacterial resistance can provide a solution to reduce the burden of this global health crisis.

Numerous studies have used immunoinformatics methods to design vaccines against various agents such as SARS CoV 2, Lassa virus (LASV), Type A influenza viruses, Saint Louis Encephalitis Virus, Echinococcus granulosus, and Sarcoptes scabiei [13, 18–26]. In this study, we aimed to design an in silico multi-epitope vaccine that affects a wide range of different bacterial NDMs. We used bioinformatics tools to find epitopes for appropriate and specific stimulation of the immune system against NDMs. An efficient vaccine should be able to stimulate CD4 ^+^ T cells and B cells, so in the current study, the vaccine candidate was designed to contain linear and discontinuous epitopes to stimulate both CD4^+^ T cells and B cells. As NDMs are active enzymes, the natural form of these proteins cannot be used due to their adverse effects on β-lactam antibiotics. On the other hand, using denatured or partially degraded forms can damage the discontinuous epitopes and hide or damage continuous epitopes. Therefore, we used a multi-epitope vaccine construct without any adverse enzymatic activity while providing the main discontinuous and continuous epitopes of the original protein.

In this study, 2194 sequences of NDM variants expressed in different pathogens were used to obtain a conserved sequence. The purpose of obtaining a conserved sequence was to have common epitopes between the NDMs of all pathogenic bacteria to have a vaccine construct with a wide range of potential applications. Continuous B cell epitope and T cell CD4 + epitopes were predicted by several servers or several algorithms and also the antigenicity and immunogenicity of these epitopes were calculated. Finally, four epitopes were selected, one for continuous B cell and three for CD4^+^ T cell (HLA-DR, HLA-DP, and HLA-DQ alleles). Also, the epitope conservancy of these four epitopes was obtained among 2194 initial sequences which demonstrated a very high conservancy of selected epitopes. Furthermore, population coverage was obtained for CD4^+^ T cell epitopes that showed a high percentage of coverage for most of the world regions including Europe, North America and, Asia.

According to the permutation of the final four epitopes, 24 different peptides were obtained that had different toxicity, allergenicity, and antigenicity. Six out of 24 primary peptides which demonstrated very high antigenicity, low toxicity, and lack of allergenicity were selected. The third structure of the six peptides was modeled after adding CTB adjuvants with an EAAAK linker and creating six different vaccines, and the discontinuous B cell epitope of all six vaccines was compared to different NDM proteins. Finally, it was observed that only in the second vaccine, there was a sequence with high similarity to a conserved discontinuous B cell epitope in original NDM proteins.

To stimulate innate immunity and prevent tolerance, the vaccine must be able to bind to innate immune receptors. Toll-like receptors (TLRs) activate innate immune cells such as neutrophils, monocytes, and dendritic cells (DCs), that lead to responses including expression of cell surface molecules and the production of cytokines. These responses contribute to the activation of the acquired immune system cells and prevent tolerance. Herein, three extracellular TLRs including TLR1, TLR1-TLR2, and TLR4 have been evaluated in the docking analysis. These TLRs detect pathogen-associated molecular patterns (PAMPs) in the vaccine adjuvant in the extracellular space. The docking of the vaccine construct with these TLRs demonstrated that they can bind through strong interactions. The predicted vaccine-TLRs complex eigenvalue was 2.129046e-05 which implies the stability of the vaccine-TLRs complex. Amino acids with correlated motions and rigid regions (stiffer springs) are present in the covariance and elastic network graphs, respectively. The presence of these amino acids implies a stable vaccine-TLR complex.

The half-life of the vaccine was estimated in both in vitro and in vivo simulations using the ProtParam tool. For example, the stability in *E. coli* was predicted to be more than 10 h, which provides the time required to extract the vaccine protein during the induction process. In addition, the vaccine construct was shown to be stable.

The results of immune simulation using the C-IMMSIM server showed that 70 days after the second dose of the NDM vaccine a much higher immune response against the NDM antigen was induced compared to a nonspecific antigen control group. In other words, the titer of antibodies increases rapidly after exposure to the specific antigen, and also the population of memory B cells, PLB cells, and T helper (Th) cells increased.

Finally, the protein sequence was Codon optimized for *E. coli* K12. The GC-Content vaccine (51.14) was very similar to the GC-Content *E. coli* K12 (50.73), which results in high-level protein expression in *E. coli*. The simulation results using the SnapGene tool confirmed that the recombinant vaccine construct can be expressed following cloning in the bacterium.

In the current study, immunoinformatic methods were used to find epitopes to design a vaccine construct with high antigenicity to specifically stimulate the immune system to prevent the NDMs adverse effects. In the current study, epitopes from several shared regions of the enzyme (NDM) were used simultaneously. The selected epitopes are highly conserved as we demonstrated their presence in all members of a collection of available sequences of NDMs including a large number of isoforms and possible mutations. Therefore, the appearance of mutations in these regions is improbable. Furthermore, as we used multiple epitopes simultaneously, the immune system of the immunized organism produces multiple antibodies against the vaccine candidate. The multiple antibodies prevent resistance even in case of mutation occurs in some of the epitopes. Indeed, this vaccine candidate mimics the combination therapy strategy regarding its resistance to antibiotics resistance. The current study demonstrated that designing a vaccine based on a conserved sequence of NDMs can stimulate immune responses efficiently. However, it is crucial to test the vaccine construct in animal and human studies to verify the results. Furthermore, the immune stimulation is not enough to approve a vaccine. It should be considered that even if the vaccine stimulates the immune system effectively, it does not necessarily mean that it can be effective in the human model. Therefore, it is obligatory to assess the efficacy and efficiency of the vaccine in both animal models and human volunteers for a long duration of time.

## Conclusion

Antibiotics resistance, especially through NDMs, is on the rise today and is referred to as a global health crisis. Although the efforts have so far focused only on developing new antibiotics, one of the best ways to avoid antibiotic resistance is preclusion through preventing excess antibiotic use or designing a resistance inhibiting vaccine. In this study, immunoinformatics strategies were used to design a vaccine against different variants of NDMs that could produce a favorable response in CD4^+^ T cells and B cells. The vaccine has also been shown to be able to bind to TLRs stably to ensure eliciting immune responses. The vaccine was also predicted to be stable and had a good half-life. The immune simulation showed that with two doses of vaccine injection a strong immune response to NDMs can be induced. Finally, the expression potential of the vaccine in the bacterial host was confirmed by simulation methods. The implementation of multiple highly conserved epitopes in the NDMs makes the vaccine candidate resistant to antibiotics resistance. However, in vivo and community-level studies are required to ensure the efficacy and efficiency of the vaccine construct.

## Methods

### Protein sequence retrieval

The complete amino acid sequences of NDM-1 to NDM-29 were retrieved from the NCBI database (https://www.ncbi.nlm.nih.gov/protein) in May 2020. The search contained enzymes with the same name but a different structure that was expressed by non-pathogenic bacteria. These non-pathogenic bacteria, namely *Kocuria rhizophila*, *Planctomycetes bacterium* Pan265, *Sphingomonadales bacterium*, and *Acinetobacter nosocomialis* were removed from the search. Furthermore, the word “cloning vector” was also removed due to the existence of a cloning vector with the same name. Finally, 2201 sequences were obtained and were aligned using Clustal Omega software by multiple sequence alignment (MSA). Furthermore, seven sequences with a large gap at the beginning or the end of the sequence were considered as partial sequences and removed from the MSA results. The NCBI accession numbers of removed partial sequences were AQT38377.1, WP_063860857.1, QID22101.1, PIL86686.1, PIL65196.1, APY22234.1, and BBE58699.1. Finally, the 2194 remaining sequences were re-aligned to obtain the conserved regions by Clustal Omega software.

### Secondary structure

Protein secondary structure including surface accessibility, Alpha helix, beta-strand, Coil, and disorder in an amino acid sequence was obtained using NetSurfP-2.0 Server (https://services.healthtech.dtu.dk/service.php?NetSurfP-2.0). NetSurfP-2.0 is a sequence-based server meaning that it can predict local structural features of proteins from the initial sequence, and “uses an architecture composed of convolutional and long short-term memory neural networks trained on solved protein structures”. The NetSurfP-2.0 server calculates second structures including helix, strand, and coil, as well as relative solvent accessibility (RSA) and disorder.

### Continuous B cell epitope

Sequential linear amino acid sequences are called continuous epitopes. Continuous B cell epitopes were retrieved from LBtope (https://webs.iiitd.edu.in/raghava/lbtope/), ABCpred (https://webs.iiitd.edu.in/raghava/abcpred/) and SVMTriP (http://sysbio.unl.edu/SVMTriP/). These three Servers work based on different algorithms. The LBtope server contains three models in three different datasets, including Fixed, Variable, and Confirm models. The Fixed model predicts only linear B-cell epitopes of 20 residues, while the Variable-model, which was used in this study, predicts variable-length B-cell epitopes. Finally, Confirm model predicts the desired continuous B cell epitopes based on experimentally validated data by two or more studies [27]. The ABCpred server uses an artificial neural network based on machine-learning technique and predicts fixed lengths of 10, 12, 14, 16, and 20 residues [28], that in the current study epitopes with 16 residues were selected. SVMTriP is based on Support Vector Machine (SVM) which works by combining Tri-peptide similarity and Propensity scores (SVMTriP) [29]. This server can also predict epitopes with lengths of 10, 12, 14, 16, and 20 residues. Herein, epitopes with a length of 16 residues were selected. The optimum epitopes were considered as the amino acid sequences standing among the top five scoring epitopes in at least one of the servers, and, the related scores stand higher than the threshold in at least one of the other two servers.

### T cell CD4^+^ epitope

The Allele Frequency Net Database (AFND) (http://www.allelefrequencies.net/hla.asp) was used to determine the frequent HLA-DP and HLA-DQ haplotypes and the HLA-DR alleles in Iran and European countries.

The T cell epitopes were predicted using the IEDB MHC II prediction tool (http://tools.immuneepitope.org/mhcii/). Consensus method was used which combines NN-align (NetMHCII 2.2), SMM-align (NetMHCII 1.1), CombLib, and Sturniolo [30]. Fifteen amino-acids length epitopes were selected. Finally, the scores obtained by the Consensus method were retrieved and the stacked column chart was drawn for HLA-DR, HLA-DP, and HLA-DQ. The length of peptides binding to MHC class II is usually between 13 and 17 amino acids, although shorter or longer length peptides can also bind to the groove of the MHC class II [31, 32], in the current study we adopted the server default length of 15 amino acids.

### Prediction of antigenicity and immunogenicity of peptide fragments

The antigenicity of epitopes obtained from B cell and T cell prediction servers was calculated by the VaxiJen v3.0 server (https://www.ddg-pharmfac.net/vaxijen3/). Unlike most methods, the VaxiJen v3.0 server uses an alignment-free approach and is based on auto-cross covariance (ACC) transformation of protein sequences into uniform equal-length vectors [33]. The T cell immunogenicity was predicted by the IEDB server (http://tools.immuneepitope.org/CD4episcore/). The IEDB recommended algorithm is a combination of the 7-allele method and the immunogenicity method, which is more accurate comparing to single methods [34].

### Epitope conservancy

The percentage of the protein sequences identity was calculated by the Epitope Conservancy tool (http://tools.iedb.org/conservancy/) to obtain identity or the degree of correspondence (similarity) of the final epitopes to the 2194 initial sequences. “Epitope linear sequence conservancy” analysis type was selected and other parameters were kept in default.

### Population coverage

T cells are only able to detect the peptide-MHC complex and therefore they only respond to the antigens when the MHC molecule can bind to fragments of antigen-related epitopes. The IEDB population coverage analysis tool (http://tools.iedb.org/population/) was used to examine the population coverage of selected T cell epitopes in the world population as well as in Iran. The selected MHC alleles were the same alleles used in T cell epitope prediction servers.

### Toxicity, allergenicity, and antigenicity of peptides

Four selected epitope fragments were permutated to obtain all possible states of the final peptide. These epitope fragments were joined by glycine-proline-rich GPGPG linkers. All permutation-derived peptides were evaluated for toxicity using the ToxDL server (http://www.csbio.sjtu.edu.cn/bioinf/ToxDL/), Allergenicity was assessed by AllerTOP v.2.0 (http://www.ddg-pharmfac.net/AllerTOP/) and AllergenFP v.1.0 (http://www.ddg-pharmfac.net/AllergenFP/) servers, and antigenicity was assessed by VaxiJen v3.0 server. ToxDL server is based on interpretable deep learning which is consists of two main components, one component is based on CNNs and the other based on multilayer perceptron with domain information [35]. Toxicity less than 0.05 was considered as a threshold. AllerTOP v.2.0 and AllergenFP v.1.0 based on auto-cross covariance (ACC) transformation of protein sequences into uniform equal-length vectors, that 2427 known allergens and 2427 non-allergens classified by the k-nearest neighbor algorithm (kNN, k = 1).

### Adjuvant and vaccine construct

Adjuvant improves immune system responses and thus leads to a strong and long-lasting response and prevents tolerance. Cholera toxin subunit B (CTB) is a non-toxic component of cholera toxin that has a high tendency to bind to the monosialotetrahexosylganglioside (GM1) receptor on immune cells such as macrophages, dendritic cells, and B cells, thereby stimulating these cells. The CTB sequence was retrieved from the Uniprot database with an entry identifier as P01556. Finally, CTB was added by the EAAAK linker to the N-terminal of peptides.

### Vaccine-structure modeling and validation

The three-dimensional (3D) structure of final vaccine candidates was modeled by the RaptorX server (http://raptorx.uchicago.edu/ContactMap/), which is a distance-based protein folding server powered by deep learning. RaptorX server provides root-mean-square deviation (RMSD) for each proposed structure in angstroms (Å), which is the measure of the average distance between the atoms for each proposed structure. The six vaccine constructs were loaded on the RaptorX server and resulted in five proposed models. Validation of the five proposed RaptorX models of each vaccine construct was performed using the MolProbity server (http://molprobity.biochem.duke.edu/), which calculated the values of favored rotamers, Ramachandran favored and Rama-Z score, and also designed the Ramachandran plot. Consequently, the top model based on MolProbity was selected for each one of the vaccine constructs.

### Discontinuous B cell epitope

Discontinuous B cell epitope Vaccines were evaluated by the ElliPro server (http://tools.iedb.org/ellipro/). Also, protein data bank (PDB) IDs of twelve different NDM proteins were extracted from the PDB database (https://www.rcsb.org/) and examined regarding Discontinuous epitopes by ElliPro server. Finally, the epitope sharing between vaccines and primary proteins was investigated and a heatmap of the final sequence was designed by R software [36].

### Molecular docking of multi-epitope vaccine with TLR receptors

To test whether the final vaccine was capable of inducing innate immunity, the innate immune receptors Toll-like receptor 1 (TLR1), TLR1-TLR2 heterodimer, TLR2, TLR4, and TLR4 / MD-2 heterodimer were docked with the vaccine candidate by PatchDock server (https://bioinfo3d.cs.tau.ac.il/PatchDock/). The docking algorithm of this server is based on Shape Complementarity Principles [37, 38]. Global binding energy that consists of attractive and repulsive van der Waals (VdW) forces, atomic contact energy (ACE), and hydrogen bond (HB) were calculated by FireDock server (https://bioinfo3d.cs.tau.ac.il/FireDock/) [39]. The top 10 results of the PatchDock server were refined by the FireDock server to rank the receptor-ligand complex based on global energy. To better verify the results, the protein-receptor complexes which were identified by FireDock as the complexes with the lowest global energy were re-checked by Molecular Mechanics-Generalized Born Surface Area (MM-GBSA) which is provided by the HawkDock server (http://cadd.zju.edu.cn/hawkdock/). Furthermore, to observe the vaccine-TLR complex interactions, the DIMPLOT program in LigPlot + software was used for visualization.

### Normal mode analysis

The iMODS server (http://imods.chaconlab.org/) was used to evaluate the stability of the vaccine-receptor complex. This server is based on Normal mode analysis (NMA) in internal (dihedral) coordinates, which can predict collective motions of macromolecules, including proteins. This server calculates deformability, B-factor, eigenvalues, variance, covariance map, and elastic network for the vaccine-receptor complex. Deformability is the ability of a molecule to deform each of its residues. The B-factors in PDB files are used to measure mobility in macromolecules, including proteins. In this server, B-factors are also derived from NMA by multiplying the NMA mobility. Eigenvalue indicates motion stiffness and its value is directly related to the deformation of the structure, i.e. the lower the value, the easier it is to deform the structure of the macromolecule. The variance is related to normal mode and is inversely related to the eigenvalue. The covariance matrix represents the bond between pairs of residues and determines the correlated, uncorrelated, or anti-correlated motions. The elastic network identifies pairs of atoms connected by a spring [40–42]. The vaccine-TLR complexes were introduced to the iMODS server. The server predicted the stability or un-stability of the complex by calculation of deformability, B-factor, eigenvalues, variance, covariance map, and elastic network for the vaccine-receptor complex and gave the results in the form of graphs.

Two main factors are required to predict the stability of molecular complex using iMODS server; coarse-grained atomic modeling and Elastic Network Mode (ENM). The coarse-grained atomic modeling has three types including CA, C5, and HA atomic models. In the current study, the CA atomic model has been used in which Cα atoms are considered as a representative of the whole residue mass. Furthermore, there are four types of ENM among which we used the edNMA model that is a molecular dynamics (MD) based ENM to predict the Cα-Cα interactions. In the edNMA model, it is not required to set any user-specified cut-off values.

### Physiochemical evaluation of vaccine

Physicochemical properties were calculated by the ProtParam tool (https://web.expasy.org/protparam/). The most important characteristic that the ProtParam tool calculates is the final vaccine construct half-live in mammalian reticulocytes and yeast and *Escherichia coli*. It also calculates other characteristics including Molecular weight, chemical formula, the total number of negatively (Asp + Glu) and positively (Arg + Lys) charged residues, theoretical isoelectric point (pI), Instability index, aliphatic index, and grand average of hydropathicity (GRAVY).

### Immune simulation

C-IMMSIM server (http://kraken.iac.rm.cnr.it/C-IMMSIM/index.php?page=1) was used to evaluate the immune stimulation and obtain immune response profile by in silico simulation. This server uses the Celada-Seiden model to obtain the immune response profile of the vaccine in the mammalian immune system [43]. Vaccine injection without LPS was selected and two doses of the vaccine were adjusted at 90 eight-hour-long steps that equal to a 30 days interval. In addition, the exposure to the NDM antigen was simulated by introducing a dose of NDM-1 300 eight-hour-long steps equals 100 days after the first dose of the final vaccine construct. Furthermore, the “RNA polymerase sigma-70 factor” was used as a non-specific antigen in a control group. The specific antigen for the vaccine was “subclass B1 metallo-beta-lactamase NDM-1” with QIV52529 accession number in the NCBI database that is expressed by the gram-negative bacterium *Klebsiella pneumoniae*. The nonspecific antigen was “RNA polymerase sigma-70 factor” which is accessible by the entry identifier A0A016CK04 in the UniProt database. This protein is expressed in the *Bacteroides fragilis* gram-negative bacterium. The Simulation period was set on 1100 eight-hour-long steps (i.e. about one year). Other parameters were set on default settings.

### Codon-optimization and cloning

The Java Codon Adaptation Tool (JCat) for codon optimization and reverse translation was used to build the cDNA sequence, which improves translation efficiency in *E. coli* K12. To express the optimized sequence of the final vaccine construct, it was inserted into the ET-28a (+) vector by the SnapGene tool (https://www.snapgene.com/try-snapgene/).

## Supplementary Information

**Additional file 1: Table 1**. Molecular docking and interaction refinement of multi-epitope vaccine with TLR receptors.

**Additional file 2: Figure 1**. Interacting residues between docked Chain V from vaccine with chain A or chain B (or both) from TLR1-TLR2 (a) and TLR4 (b, c). The green, red, and brick red dashed lines represent hydrogen bonds, salt bridges, and hydrophobic interactions, respectively.

**Additional file 3: Figure 2**. Molecular dynamics simulation of the vaccine with TLR1-TLR2 (a) and TLR4 (b, c) complex. The graphs represent (I) Deformability, (II) B-factor, (III) Eigenvalues, (IV) Variance, (V) Covariance map (VI) Elastic network.

## Data Availability

All the data supporting the findings are contained within the manuscript.

## Abbreviations

NDM: New Delhi metallo-beta-lactamase
TLR: Toll-like receptors
RSA: Relative solvent accessibility
RMSD: Root-mean-square deviation
SVM: Support vector machine
MSA: Multiple sequence alignment
AFND: Allele frequency net database
ACC: Auto-cross covariance
CTB: Cholera toxin subunit B
GM1: Monosialotetrahexosylganglioside
VdW: Van der Waals
ACE: Atomic contact energy
HB: Hydrogen bond
MM-GBSA: Molecular mechanics-generalized born surface area
NMA: Normal mode analysis
GRAVY: Grand average of hydropathicity
