# Supplementary material for: In silico vaccine design and epitope mapping of New Delhi metallo-beta-lactamase (NDM): an immunoinformatics approach
Source: BMC Bioinformatics. 2021 Sep 25;22:458. doi: 10.1186/s12859-021-04378-z (PMC8465709; doi:10.1186/s12859-021-04378-z)
Supplement: Supplementary file 1 — Additional file 1: Table 1. Molecular docking and interaction refinement of multi-epitope vaccine with TLR receptors. [file 12859_2021_4378_MOESM1_ESM.docx]

| **Supplementary table 1. Molecular docking and interaction refinement of multi-epitope vaccine with TLR receptors.** | | | | | | | |
| --- | --- | --- | --- | --- | --- | --- | --- |
|  | **PatchDock** | | **FireDock** | | | | |
|  | **Solution No** | **Score** | **Global Energy** | **Attractive VdW** | **Repulsive VdW** | **ACE** | **HB** |
| **TLR1**  **(6nih)** | **1** | **19836** | **137.06** | **-26.41** | **204.35** | **0.73** | **-2.45** |
|  | **2** | **16298** | **28.17** | **-9.47** | **2.73** | **6.06** | **-0.99** |
|  | **3** | **16198** | **694.41** | **-21.79** | **872.01** | **16.15** | **-2.91** |
|  | **4** | **16014** | **9.94** | **-1.55** | **0.11** | **3.45** | **0.00** |
|  | **5** | **15840** | **0.90** | **-10.35** | **5.13** | **4.17** | **-2.64** |
|  | **6** | **15622** | **-5.75** | **-17.91** | **15.91** | **7.74** | **-1.54** |
|  | **7** | **15564** | **-8.98** | **-12.23** | **1.35** | **8.24** | **-3.38** |
|  | **8** | **15408** | **21.74** | **-18.00** | **5.32** | **13.72** | **-0.57** |
|  | **9** | **15394** | **9.73** | **-23.77** | **6.38** | **16.96** | **-2.58** |
|  | **10** | **15210** | **-34.76** | **-39.92** | **19.23** | **12.33** | **-1.74** |
| **TLR1-TLR2 (2z7x)** | **1** | **18332** | **59.80** | **-54.72** | **180.96** | **13.56** | **-10.92** |
|  | **2** | **16938** | **786.55** | **-66.97** | **1047.44** | **25.22** | **-10.75** |
|  | **3** | **16836** | **178.31** | **-32.68** | **223.95** | **17.99** | **-4.20** |
|  | **4** | **16800** | **6.52** | **-20.73** | **7.65** | **17.18** | **-2.23** |
|  | **5** | **16454** | **9.79** | **-0.00** | **0.00** | **0.00** | **0.00** |
|  | **6** | **16420** | **3.33** | **-12.87** | **1.70** | **9.38** | **-2.53** |
|  | **7** | **16344** | **-14.24** | **-19.25** | **3.87** | **4.89** | **-2.11** |
|  | **8** | **15798** | **20.58** | **-4.11** | **1.92** | **4.44** | **0.00** |
|  | **9** | **15530** | **12.55** | **-5.64** | **3.86** | **6.83** | **0.00** |
|  | **10** | **15278** | **52.78** | **-23.04** | **65.17** | **13.69** | **-5.87** |
| **TLR1-TLR2 (2z80)** | **1** | **15092** | **-3.00** | **-35.40** | **18.27** | **13.40** | **-3.11** |
|  | **2** | **14740** | **11.94** | **-1.00** | **0.15** | **1.03** | **0.00** |
|  | **3** | **14676** | **-3.25** | **-22.60** | **8.49** | **2.83** | **-4.22** |
|  | **4** | **14342** | **0.56** | **-22.74** | **21.20** | **14.78** | **-4.65** |
|  | **5** | **14222** | **4.78** | **-29.54** | **17.41** | **14.81** | **-3.93** |
|  | **6** | **14140** | **-1.27** | **-26.76** | **12.27** | **13.60** | **-4.50** |
|  | **7** | **14090** | **-39.00** | **-34.48** | **10.60** | **-2.98** | **-5.48** |
|  | **8** | **14066** | **47.68** | **-15.54** | **6.67** | **10.95** | **0.00** |
|  | **9** | **14020** | **6.00** | **-3.14** | **0.34** | **2.39** | **0.00** |
|  | **10** | **13924** | **69.96** | **-39.95** | **104.47** | **18.74** | **-4.17** |
| **TLR4**  **(2Z62)** | **1** | **14908** | **-35.78** | **-37.75** | **21.82** | **16.94** | **-3.54** |
|  | **2** | **14830** | **540.49** | **-26.99** | **700.21** | **9.16** | **-3.67** |
|  | **3** | **13700** | **20.95** | **-33.98** | **29.75** | **20.01** | **-5.17** |
|  | **4** | **13212** | **1622.93** | **-43.21** | **2100.00** | **4.99** | **-3.31** |
|  | **5** | **13208** | **1.95** | **-40.66** | **39.97** | **19.69** | **-8.50** |
|  | **6** | **13060** | **4.94** | **-19.05** | **4.69** | **10.45** | **-0.98** |
|  | **7** | **13056** | **-45.30** | **-47.09** | **33.03** | **11.64** | **-3.63** |
|  | **8** | **12990** | **32.62** | **-25.82** | **9.75** | **19.27** | **-1.61** |
|  | **9** | **12990** | **18.36** | **-18.60** | **9.95** | **17.28** | **-3.88** |
|  | **10** | **12968** | **22.27** | **-22.08** | **6.39** | **22.07** | **-5.25** |
| **TLR4**  **(2z63)** | **1** | **16704** | **4395.44** | **-45.35** | **5580.54** | **3.36** | **-7.85** |
|  | **2** | **15838** | **54.48** | **-40.51** | **135.40** | **14.79** | **-5.36** |
|  | **3** | **15746** | **1389.37** | **-26.75** | **1759.89** | **3.60** | **-3.40** |
|  | **4** | **15652** | **10260.89** | **-83.04** | **12926.38** | **26.44** | **-14.26** |
|  | **5** | **15580** | **11.50** | **-10.11** | **6.54** | **5.06** | **-0.54** |
|  | **6** | **15574** | **3755.68** | **-67.64** | **4840.49** | **7.70** | **-10.67** |
|  | **7** | **15460** | **-5.43** | **-8.97** | **3.69** | **1.12** | **-0.80** |
|  | **8** | **15344** | **223.90** | **-21.45** | **268.50** | **10.20** | **-1.80** |
|  | **9** | **15316** | **21.37** | **-7.91** | **0.52** | **7.28** | **-0.81** |
|  | **10** | **15060** | **23.80** | **-39.28** | **10.84** | **29.00** | **-5.05** |
| **TLR4-MD-2 (3fxi)** | **1** | **18718** | **1.98** | **-0.91** | **0.00** | **-0.45** | **0.00** |
|  | **2** | **17914** | **589.40** | **-65.79** | **837.81** | **17.01** | **-9.60** |
|  | **3** | **17536** | **5.24** | **-0.81** | **0.00** | **0.89** | **0.00** |
|  | **4** | **17104** | **-0.61** | **-13.97** | **3.91** | **6.02** | **-0.89** |
|  | **5** | **16768** | **287.10** | **-30.65** | **352.50** | **22.99** | **-1.77** |
|  | **6** | **16636** | **1570.51** | **-27.74** | **1978.28** | **13.00** | **-7.85** |
|  | **7** | **16560** | **2182.88** | **-57.38** | **2830.90** | **5.02** | **-7.31** |
|  | **8** | **16356** | **361.02** | **-36.20** | **470.42** | **12.62** | **-7.78** |
|  | **9** | **16322** | **5.98** | **-21.67** | **3.21** | **11.83** | **-1.96** |
|  | **10** | **16000** | **8.50** | **-5.40** | **10.03** | **0.93** | **-0.58** |
