# Supplementary figures and images for: In silico vaccine design and epitope mapping of New Delhi metallo-beta-lactamase (NDM): an immunoinformatics approach
Source: BMC Bioinformatics. 2021 Sep 25;22:458. doi: 10.1186/s12859-021-04378-z (PMC8465709; doi:10.1186/s12859-021-04378-z)

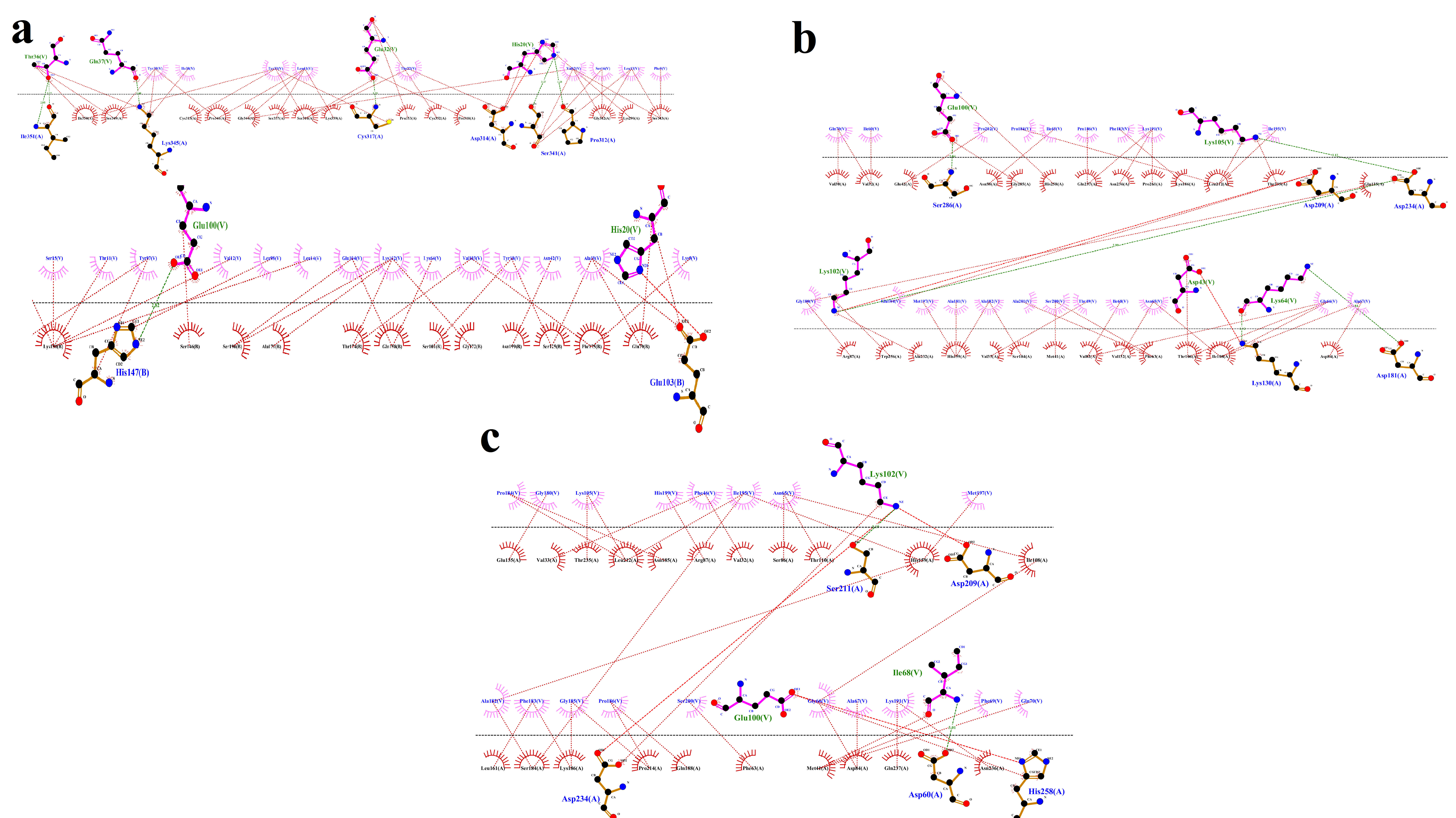

Supplement: Supplementary file 2 — Additional file 2: Figure 1. Interacting residues between docked Chain V from vaccine with chain A or chain B (or both) from TLR1-TLR2 (a) and TLR4 (b, c). The green, red, and brick red dashed lines represent hydrogen bonds, salt bridges, and hydrophobic interactions, respectively. [file 12859_2021_4378_MOESM2_ESM.png]

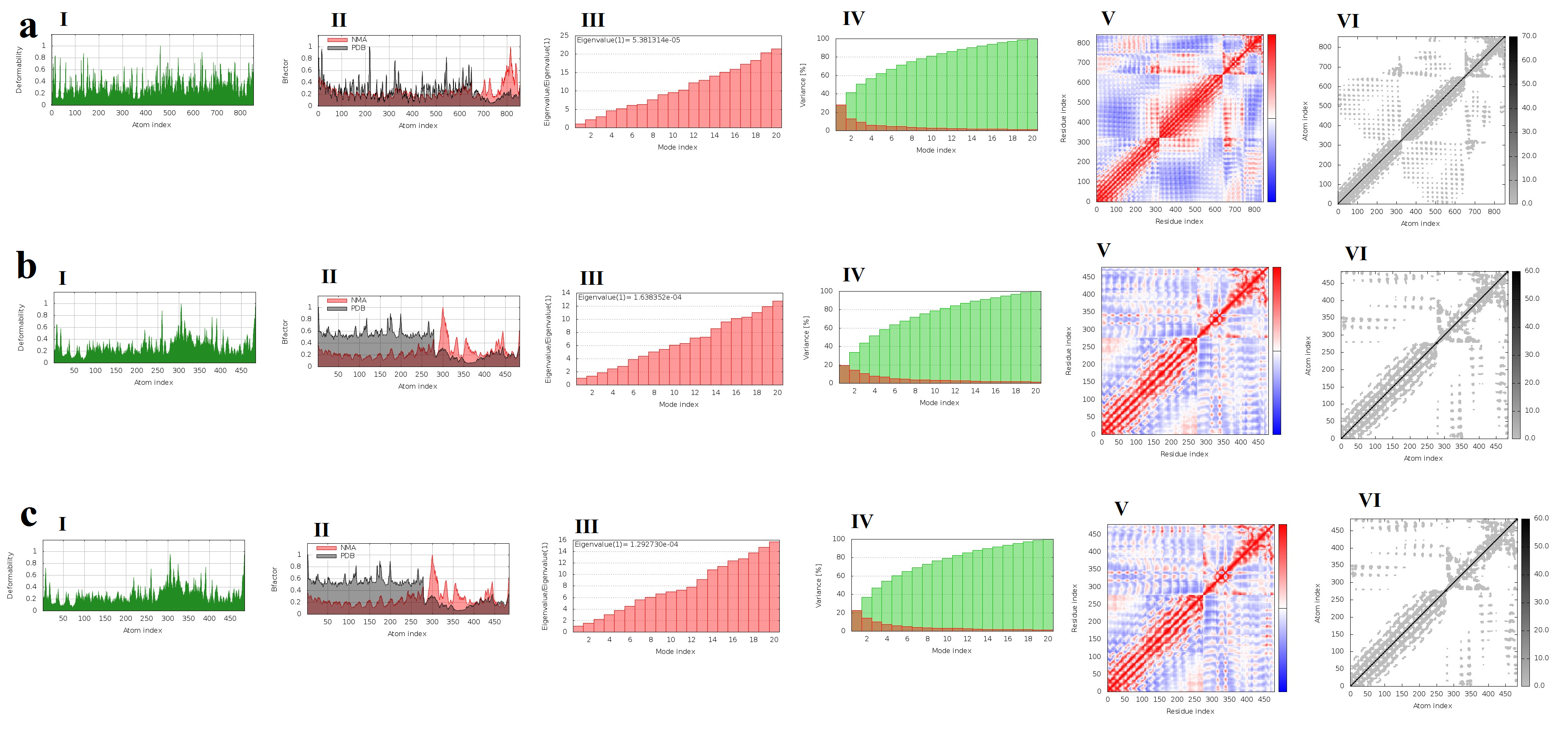

Supplement: Supplementary file 3 — Additional file 3: Figure 2. Molecular dynamics simulation of the vaccine with TLR1-TLR2 (a) and TLR4 (b, c) complex. The graphs represent (I) Deformability, (II) B-factor, (III) Eigenvalues, (IV) Variance, (V) Covariance map (VI) Elastic network. [file 12859_2021_4378_MOESM3_ESM.png]
